# Chloroplasts Isolation from *Chlamydomonas reinhardtii* under Nitrogen Stress

**DOI:** 10.3389/fpls.2017.01503

**Published:** 2017-08-29

**Authors:** Miao Yang, Jun-Peng Jiang, Xi Xie, Ya-Dong Chu, Yan Fan, Xu-Peng Cao, Song Xue, Zhan-You Chi

**Affiliations:** ^1^Marine Bioengineering Group, Dalian Institute of Chemical Physics, Chinese Academy of Sciences Dalian, China; ^2^University of Chinese Academy of Sciences Beijing, China; ^3^School of Life Sciences and Biotechnology, Dalian University of Technology Dalian, China; ^4^Liaoning Ocean and Fisheries Science Research Institute Dalian, China

**Keywords:** *Chlamydomonas*, chloroplast isolation, triacylglycerol, nitrogen stress, fatty acid biomarkers

## Abstract

Triacylglycerols are produced in abundance through chloroplast and endoplasmic reticulum pathways in some microalgae exposed to stress, though the relative contribution of either pathway remains elusive. Characterization of these pathways requires isolation of the organelles. In this study, an efficient and reproducible approach, including homogenous batch cultures of nitrogen-deprived algal cells in photobioreactors, gentle cell disruption using a simple custom-made disruptor with mechanical shear force, optimized differential centrifugation and Percoll density gradient centrifugation, was developed to isolate chloroplasts from *Chlamydomonas reinhardtii* subjected to nitrogen stress. Using this approach, the maximum limited stress duration was 4 h and the stressed cells exhibited 19 and 32% decreases in intracellular chlorophyll and nitrogen content, respectively. Chloroplasts with 48 – 300 μg chlorophyll were successfully isolated from stressed cells containing 10 mg chlorophyll. These stressed chloroplasts appeared intact, as monitored by ultrastructure observation and a novel quality control method involving the fatty acid biomarkers. This approach can provide sufficient quantities of intact stressed chloroplasts for subcellular biochemical studies in microalgae.

## Introduction

The chloroplast is a specialized subcellular compartment of plant and algal cells that is derived from cyanobacteria through endosymbiosis (Moreira et al., 2000; Okamoto and Inouye, 2005). Through the process of photosynthesis, this organelle converts solar energy into biochemical energy, capturing environmental CO_2_ and releasing O_2_ (Engel et al., 2015). In addition to photosynthesis, multiple other processes occur in chloroplast, including biosyntheses of chlorophyll (Chl), amino acids, and fatty acids as well as membrane lipid assembly and trafficking (Baginsky and Gruissem, 2004; Block and Jouhet, 2015). Furthermore, chloroplast degradation and turnover and recycling of chloroplast nutrients play a crucial role in response to adverse environmental conditions, which is required to meet energetic demands and maintain homeostasis (Xie et al., 2015). Although chloroplasts have been isolated from plants for analyses of tolerance to oxidative stresses, such as chilling, heat, osmotic and salt stress (Robinson et al., 1983; Heckathorn et al., 1998; Ling and Jarvis, 2016; Meng et al., 2016), such mechanistic studies in algae have been hampered by unavailable protocols for chloroplast isolation from stress-induced cells, especially microalgae.

In the past decade, *Chlamydomonas reinhardtii* has emerged as a model green microalga for investigations of many aspects of lipid metabolism (Li-Beisson et al., 2015), especially biosynthesis of the promising biofuel feedstock triacylglycerol (TAG) (Ruiz et al., 2016; Zienkiewicz et al., 2016). Increasing evidences reveal that parallel TAG biosynthesis pathways in the chloroplast and endoplasmic reticulum (ER) concomitantly exist in microalgae, especially the plastid pathways including *de novo* TAG biosynthesis and turnover of chloroplast membrane lipids into TAG (Goodson et al., 2011; Legeret et al., 2015; Goold et al., 2016; Xu et al., 2016; Balamurugan et al., 2017), which are distinct from the relatively known pathways present in higher plants and yeast (Xu et al., 2016). However, the specific contribution of the chloroplast and ER to TAG assembly remains ambiguous (Zienkiewicz et al., 2016). To date, there are abundant studies in *C. reinhardtii* involving the chloroplast than ER under normal growth conditions (Mason et al., 2006). As the chloroplastidic lipid remodeling that occurs in *C. reinhardtii* following stress conditions, nitrogen deprivation in particular, is proposed to be closely correlated to TAG synthesis (Li-Beisson et al., 2015; Du and Benning, 2016; Xu et al., 2016), it is essential to obtain stressed chloroplasts from this alga. Despite a brief mention by Fan et al. (2011), without any data presented, chloroplast isolation from stressed *C. reinhardtii* has yet to be achieved.

In general, the entire process of chloroplast isolation from microalgae consists of four major steps: algal cultivation, cell disruption, chloroplast purification and quality evaluation (Mason et al., 2006). In the past 50 years, distinct isolation methods have been employed to purify *C. reinhardtii* chloroplasts in different physiological states for different research purposes, including studies of photosynthetic activity (Klein et al., 1983; Mendiola-Morgenthaler et al., 1985), transport studies (Moroney et al., 1987; Mason et al., 1990) and metabolic enzyme characterization (Bollivar and Beale, 1995; Jans et al., 2008; Burgess et al., 2016). Nonetheless, these chloroplasts were all obtained from normally growing algal cells. Hence, there is a need to isolate intact chloroplasts from stressed *C. reinhardtii* cells to investigate the dynamic response of this crucial organelle to abiotic stress at a subcellular level; relevant analyses include characterization of subcellular TAG biosynthesis, remodeling of the chloroplastidic glycerolipidome and omics studies of chloroplastidic lipid droplets in microalgae. Regardless, it is well known that isolation of stressed chloroplasts is difficult due to the fact that chloroplasts undergo gradual degradation in response to stress (Xie et al., 2015; Zienkiewicz et al., 2016), which is characterized by disorganization of thylakoid membranes and accumulation of starch granules and lipid droplets (Warakanont et al., 2015). These alterations severely distort the microalgal chloroplast membrane structure (Fan et al., 2011; Preininger et al., 2015), similar to what occurs in plants (Meng et al., 2016; Woodson, 2016). Thus, it is difficult to ensure the integrity of stressed chloroplasts during isolation.

To address this issue, we developed a protocol for isolating nitrogen-stressed chloroplasts from *C. reinhardtii*. The protocol includes homogenous batch cultivation in PBRs with the maximum limited stress duration, gentle cell disruption with the aid of a custom-made disruptor, optimized purification involving centrifugations and novel quality assessment based on the fatty acid biomarker analysis.

## Materials and Methods

### Microalgal Strains and Pre-cultures

A cell wall-less mutant of *C. reinhardtii* strain CC4326 was obtained from the Chlamydomonas Resource Center^1^. The cells were first grown on agar-solidified minimal medium (Mason et al., 2006) for 2 months at 50 μmol m^-2^ s^-1^ at 25°C. Algal patches were then transferred to 50 ml liquid minimal medium for 7 days to produce sufficient biomass (∼ 1 × 10^6^ cells ml^-1^) as a pre-inoculation culture, which was designated the 1^st^generation culture. The light cycle was set as 12 h light/12 h dark. The liquid cultures were centrifuged and added to fresh medium as the 2nd generation for another 7 days. Until the 4th generation, synchronous cultures were transferred on day 4 to photobioreactors (PBRs) at an initial cell density of ∼10^4^ cells ml^-1^. The 5th, 6th, and up to 10th generations were used as candidates for batch cultures for chloroplast isolation.

### Batch Cultures in PBRs under Normal and Stress Conditions

Algal cultures were normally grown in minimal medium in PBRs (4.5 cm in diameter, 45 cm in height, 600 ml culture volume), bubbled with air (120 ml min^-1^) containing 4% (v/v) CO_2_ and illuminated from one side by cool white fluorescent tubes. Each experiment was independently repeated at least three times.

Pre-inoculated cells were utilized for two-stage cultures, including nitrogen-replete conditions for the first 4 days (the first 2 days under continuous illumination and the last 2 days under a 12-h light/12-h dark cycle) under a light intensity of 50 μmol m^-2^ s^-1^ followed by nitrogen-deprived conditions for 4 h at an irradiance of 100 μmol m^-2^ s^-1^. The nitrogen starvation time was limited to 4 h based on prior experiments. N-replete cells were harvested at 4 h into the third light cycle by centrifugation for 5 min at 3000 × *g* and 4°C. A proportion of cultures with aliquots of cells containing 10 mg Chl were used for isolation of normal chloroplasts. The remaining pellet was washed once with nitrogen-free minimal medium using a fine paint brush, centrifuged as above, and transferred to the same medium before adjusting the optical density to ∼0.752. The cells were gradually stress-induced and sampled every hour for subsequent analysis. After 4 h of nitrogen deprivation, all the cells were collected (3000 × *g*, 5 min, 4°C) for isolating stress-induced chloroplasts.

### Growth Measurement, Chl Quantification and Elemental Analysis

Cellular growth parameters, including optical density at 750 nm and cell concentration, were monitored using a spectrophotometer (Jasco v-530, Japan) and a hemacytometer, respectively. To determine biomass, ∼5 ml of culture were filtered onto a pre-weighed Whatman GF/C filter (47 mm diameter) and dried to a constant weight with a net biomass of more than 2 mg at 60°C. The illumination intensity was determined using a photosynthetically active radiation (PAR) detector (Optometer P9710 with PAR detector 3701, Gigahertz Optik Corporation, Germany). The photosystem II quantum yield, F_v_/F_m_, of the cells was measured using a Chl fluorometer (Water-PAM WALZ, Germany) according to the methods described by Yao et al. (2012).

For Chl determination, cells in 2 ml of culture were pelleted, followed by sonication in 2 ml of ethanol on ice. The pigment extracts were centrifuged at 8000 × *g* for 2 min, and the absorption of the supernatant was determined spectrophotometrically at 649 and 665 nm. The Chl concentration was calculated as Chl = 18.08 OD_649_ + 6.63 OD_665_, according to the methods of Jespersen and Christoffersen (1987).

Quantification of microagal cell nitrogen was performed using a vario EL cube elemental analyzer (Elementar, Germany). 2∼4 mg of lyophilized biomass were accurately weighed using a Mettler Toledo XP6 microbalance (Mettler Toledo GmbH, Germany).

### Chloroplast Isolation under Normal and Stress Conditions

**FIGURE 1:**
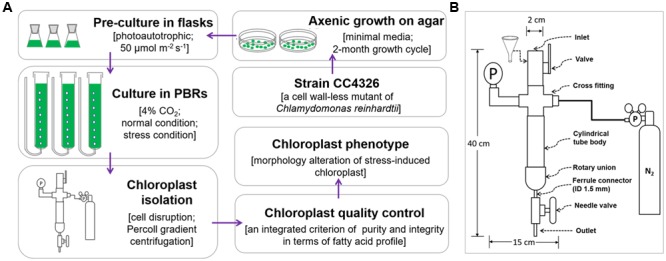
The flow chart of chloroplast isolation from *C. reinhardtii*. **(A)** The whole process for chloroplast isolation from *C. reinhardtii*. **(B)** The structure diagram of the custom-made cell disruptor. ID means the inner diameter.

All chloroplast isolation procedures were conducted at 4°C or on ice using a modified version (**Figure 1A**) of Mason et al.’s (2006) protocol. A fine paint brush was used to completely resuspend pelleted cells, and all centrifugations were performed using swing bucket rotors. Normally cultured cells with 10 mg Chl each were washed twice with 50 ml 50 mM HEPES-KOH (pH 7.5) (3000 × *g*, 5 min) to remove the small gelatinous particles surrounding the cells (Goyal et al., 1988) and to prevent the chloroplasts from clumping. Next, another 4 ml of wash buffer were used to resuspend the pellets. A custom-made disruptor (**Figure 1B**) of stainless steel was used to break the cells. From top to bottom, the device was composed of an inlet, a valve, a cross fitting, a pressure gage, a cylindrical tube body, a rotary union, a ferrule connector (inner diameter, 1.5 mm), a needle valve and an outlet. The cross fitting was connected to a standard high-pressure nitrogen tank equipped with a pressure gauge and a decompression valve. Before breaking the cells, the gas pressure was adjusted to the designated value (0.35, 0.55 or 0.75 MPa, **Tables 1, 3**). Once the cell disruptor was prepared, 20 ml isolation buffer (0.3 M sorbitol, 50 mM HEPES-KOH [pH 7.5], 2 mM Na_2_-EDTA [pH 8.0], 1 mM MgCl_2_, 1% bovine serum albumin (BSA)) were added to the cell concentrates followed by prompt injection into the cylindrical tube body with the aid of a funnel (**Figure 1B**). The concentrated cells were disrupted by rapid extrusion through the cell disruptor under distinct pressures (0.35, 0.55, or 0.75 MPa, **Tables 1, 3**). The flow rate was manually manipulated at ∼0.1 ml s^-1^, and the pressates were collected into a 50-ml ice-cold centrifuge tube.

**Table 1:** Distinct yields and fatty acid methyl ester mass ratio (R) of chloroplasts isolated from *C. reinhardtii* under normal conditions.

Group	Condition	Yield (%)	*R*
N + 1	0.35 MPa; 750 × *g*, 2 min; 670 × *g*, 1 min	14 ± 1	7.36 ± 0.25
N + 2	0.35 MPa; 5000 × *g*, 2 min; 5000 × *g*, 1 min	30 ± 2	8.91 ± 0.34
N + 3	0.55 MPa; 750 × *g*, 2 min; 670 × *g*, 1 min	16 ± 2	7.22 ± 0.44
N + 4	0.55 MPa; 5000 × *g*, 2 min; 5000 × *g*, 1 min	26 ± 2	8.91 ± 0.43
N + 5	0.75 MPa; 750 × *g*, 2 min; 670 × *g*, 1 min	13 ± 1	7.54 ± 0.51
N + 6	0.75 MPa; 5000 × *g*, 2 min; 5000 × *g*, 1 min	28 ± 2	9.89 ± 0.50
N + cell	nd	nd	4.73 ± 0.27

*N + 1, N + 2, N + 3, N + 4, N + 5, and N + 6 refer to normal chloroplasts isolated under six conditions. N + cell refers to cells cultured under normal conditions. The yield is calculated as “(chlorophyll content of isolated chloroplasts)/(chlorophyll content of the original cells) × 100%”. *R* means the mass ratio of (16:4n3 + 18:3n3) to (18:3n6 + 18:4n3) and is calculated using the data of **Figure 4A**. nd, not detected. Data are mean ± standard deviation of three measurement replicates from one independent experiment (*n* = 3).*

Several centrifugation conditions were tested to pellet chloroplasts (**Tables 1, 3**). Every ∼12 ml of the pressates were centrifuged (750 or 5000 × *g*, 2 min) to pellet chloroplasts, whole cells and cell fragments. The pellet was resuspended in 2 ml chloroplast isolation buffer and then layered onto a discontinuous Percoll gradient (20%/45%/65%) followed by centrifugation at 4200 × *g* for 15 min. The chloroplast band between the 45 and 65% layers was pipetted into another round-bottom centrifuge tube, diluted with 10 ml chloroplast isolation buffer, and centrifuged at 670 or 5000 × *g* for 1 min to remove the Percoll or small particulates. The chloroplast pellet was finally resuspended in ≥250 μl of 50 mM HEPES-KOH (pH 8.0) and 0.3 M sorbitol. The collected chloroplast fraction was then used for Chl determination and subsequent analysis.

### Fatty Acid Composition Analysis

Quantification of fatty acids was determined using one-step acid-catalyzed direct transesterification (Liu et al., 2015). Fresh cells or chloroplasts of no less than 300 μg dry biomass were transferred to a 10-ml flask; 5 ml 2% H_2_SO_4_-methanol (v/v, H_2_SO_4_/methanol) were added, and the mixtures were stirred at 70°C for 1 h with refluxing. After the mixtures were cooled to room temperature, 2 ml of hexane and 0.75 ml of distilled water were added, and then the mixtures were vortexed for 30 s. The upper hexane layer with fatty acid methyl esters (FAMEs) was analyzed by gas chromatography (Agilent 7890A GC) using triheptadecanoin as an internal standard. When necessary, the hexane extracts were concentrated under N_2_. The mass of chloroplast fatty acid was normalized to the original cellular dry biomass, which was based on the Chl amount, and denoted as mg of fatty acids per g of the equivalent cellular dry biomass.

### Immunoblotting Analysis

To ascertain the purity of the *C. reinhardtii* chloroplast preparation, each chloroplast fraction was tested for enrichment of specific marker proteins. Due to our focus on a pure chloroplast fraction free of ER, we used an ER marker protein, binding immunoglobulin protein (BIP) (Warakanont et al., 2015; Liu et al., 2016), to evaluate the purity. All chloroplast fractions were processed by immunoblotting, as described below. Fresh cells or chloroplast samples were initially resuspended in protein extraction buffer [50 mM Tris-HCl, pH 7.5, 0.15 M NaCl, 1 mM Na_2_-EDTA, 1% NP-40, 10% glycerol and 1 mM phenylmethylsulfonyl fluoride (PMSF)] and then sonicated on ice to completely lyse the cells or chloroplasts. The lysate was centrifuged at 12,000 × *g* for 15 min at 4°C. The pellets were discarded, and the extracted proteins from equal amounts of cells and chloroplasts (3 × 10^6^ cells or chloroplasts) were loaded and separated by 10% sodium dodecyl sulfate polyacrylamide gel electrophoresis (SDS-PAGE). The proteins were electroblotted onto polyvinylidene fluoride (PVDF) membranes in transfer buffer (60 mM Tris, 48 mM glycine, 1.6 mM SDS and 20% methanol) for 2 h at 200 mA, and the membranes were blocked for 2 h in phosphate-buffered saline/Tween 20 (PBST; 1.5 mM KH_2_PO_4_, 8 mM Na_2_HPO_4_, 0.1 M NaCl, 3 mM KCl, 0.05% Tween 20, v/v) with 5% (w/v) non-fat dried milk. The membranes were washed with PBST at room temperature for 5 min and then incubated overnight at 4°C with a specific primary antibody (SC-33757 Santa Cruz Biotechnology, Inc.,)^2^ at a 1:200 dilution. Following equilibration for 1 h at room temperature, the incubated membranes were then washed three times with PBST at 15 min intervals. Goat anti-rabbit secondary antibodies conjugated with horseradish peroxidase (A0208 Beyotime Institute of Biotechnology^3^) at a 1:1000 dilution were incubated with the blots at room temperature for 2 h, followed by washing as described above. The blots were developed using DAB Horseradish Peroxidase Color Development Kit (P0203 Beyotime Institute of Biotechnology) for 15 min in the dark at room temperature.

### Transmission Electron Microscopy

**FIGURE 2:**
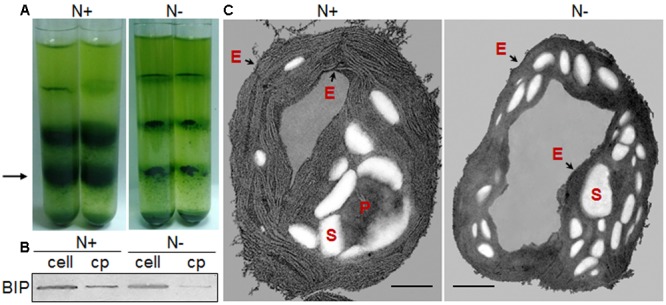
Chloroplast morphology of *C. reinhardtii* in response to nitrogen stress. **(A)** Bands of chloroplasts in a Percoll gradient. A 3-ml aliquot of cell pressate was layered onto a discontinuous Percoll gradient, with 3 ml each of 20, 45, and 65%, top to bottom. Chloroplasts predominated at the 45–65% interface (black arrow). **(B)** Chloroplast fractions (cp) were compared with the whole-cell lysates (cell) based on an endoplasmic reticulum (ER) marker protein, binding immunoglobulin protein (BIP). **(C)** Ultrastructural alterations of the chloroplast following nitrogen starvation, showing the starch granules (S), pyrenoid (P) and the chloroplast envelope membrane (black arrows - E). Scale bar = 500 nm. N+ represents Group N + 6 under normal conditions; N- represents Group N-5 under nitrogen stress.

For transmission electron microscopy, pelleted chloroplasts or cells were fixed overnight in 2.5% (v/v) glutaraldehyde in chloroplast isolation buffer for chloroplasts or HEPES-KOH (pH 7.5) buffer for cells. After the cells and chloroplasts were embedded in 2% agarose, the samples were washed with the appropriate buffer three times at 15-min intervals and post-fixed for 2 h in 1% osmic acid (w/v) at 4°C. Following the washing procedure described above, the samples were dehydrated through an alcohol series (50∼90% alcohols) for 15 min each and finally rinsed 3 times with 100% ethanol at 10-min intervals. The samples were slowly infiltrated at 4°C with epoxy propane and epoxy resin for several changes and polymerized for 12 h at 37°C, for 24 h at 25°C and for 24 h at 60°C. Ultrathin sections were stained with 2% uranyl acetate for 30 min and lead citrate for 15 min and then observed under JEM-1200EX (**Figure 2C**, N+) and JEM-2100 (**Figure 2C**, N-) electron microscopes (JEOL Ltd., Tokyo, Japan) operated at 120 kV. Micrographs were obtained using an ANT camera system.

## Results

### Chloroplast Isolation from *C. reinhardtii* under Normal Conditions

**FIGURE 3:**
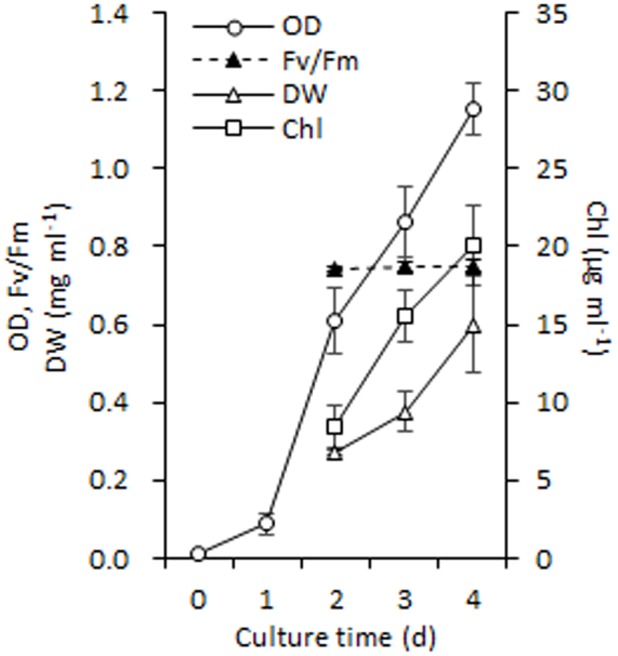
Growth performance of *C. reinhardtii* in a photobioreactor under normal conditions. Data are the means of three independent experiments (*n* = 3). Error bars represent standard deviations. OD, Fv/Fm, DW and Chl refer to the optical density at 750 nm, photosystem II quantum yield, cellular dry weight and chlorophyll.

It is necessary to obtain sufficient amounts of homogenous cells before isolating chloroplasts from *C. reinhardtii* and thus, the synchronous batch cultures in PBRs were performed (see Materials and methods). These fully synchronized algal cells grew to mid-log phase after a normal culture of 100 h in PBRs (**Figure 1A**), during which the cell density increased from ∼ 10^4^ to 10^7^ cells ml^-1^ (the OD_750_ increased from 0.01 to 1.15) and F_v_ /F_m_ (photosystem II quantum yield) was maintained at 0.747 ± 0.003 (**Figure 3**). In addition, the cell dry weight density and Chl concentration increased to 0.6 mg ml^-1^ and 20.11 μg ml^-1^, respectively (**Figure 3**). At this time point, the algal cells were vigorous and sufficient enough for chloroplast isolation.

Further, Percoll discontinuous gradient centrifugation generated three layers between distinct gradients. Microscopy observation revealed that bands between 20 and 45% contained broken chloroplasts, e.g., thylakoid membranes, that bands between 45 and 65% contained mostly intact chloroplasts, and that bands below 65% contained unbroken cells (**Figure 2A**); these results agreed with the previous report (Mason et al., 2006). During the initial experiments, distinct disruption pressures resulted in distinct patterns of fragmentation, and some chloroplasts occurred in the upper band under distinct centrifugation conditions. To obtain high-quality chloroplasts with a high yield, purity and integrity, six conditions for chloroplast isolation were tested, including three disruption pressures and two centrifugation speeds for collecting pressates and chloroplasts (**Table 1**). Starting with a 500 ml uniform culture containing 10 mg Chl, the chloroplast yields under all six conditions ranged between 13 and 30% based on Chl recovery (**Table 1**). Moreover, the chloroplast yield under the higher rotation speed (5000 × *g* for 2 min, 5000 × *g* for 1 min, **Table 1**) was approximately 2- fold higher than that under the lower speed (750 × *g* for 2 min, 670 × *g* for 1 min, **Table 1**) at the same disruption pressure. Within range of disruption pressures from 0.35 to 0.75 MPa, the higher speed could yield more amounts of chloroplasts.

**FIGURE 4:**
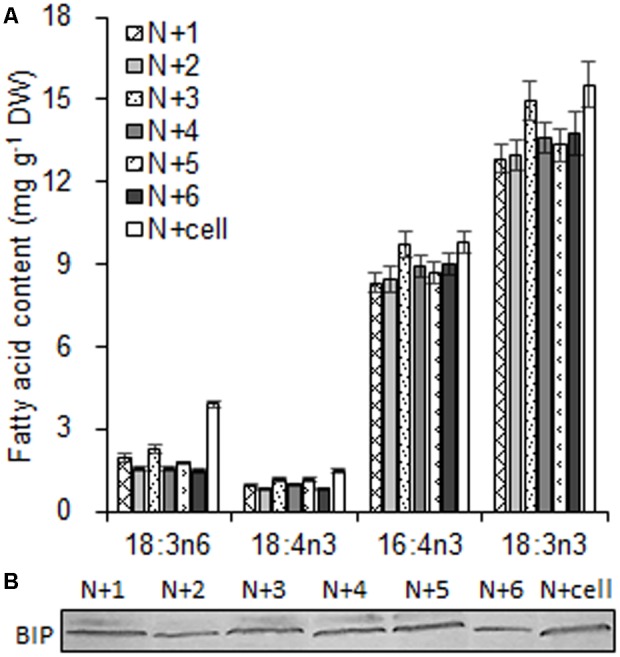
The fatty acid biomarkers and immunoblotting analysis of chloroplasts isolated from *C. reinhardtii* under normal conditions. **(A)** The fatty acid biomarkers of chloroplasts was compared to that of the whole cells. **(B)** Western blotting for an endoplasmic reticulum (ER) marker protein, binding immunoglobulin protein (BIP), was conducted to detect ER contamination of the chloroplast fraction. N + 1, N + 3, and N + 5 refer to normal chloroplasts isolated after exposure to 0.35, 0.55, and 0.75 MPa, respectively, under the same rotation speeds of 750 g and subsequently 670 g. N + 2, N + 4, and N + 6 referred to normal chloroplasts isolated from 0.35, 0.55, and 0.75 MPa, respectively, under the same rotation speeds of 5000 × g and subsequently 5000 × *g*. N + cell refers to cells cultured under normal conditions. The cellular fatty acid content is denoted as mg of fatty acyl groups per g of the cellular biomass (DW). The mass of chloroplast fatty acid was normalized to the original cellular dry biomass, which was based on the chlorophyll (Chl) amount, and denoted as mg of fatty acids per g of the equivalent cellular dry biomass. The fatty acyl is expressed as number of carbons:number of double bonds with n-number, indicating the position of the double bond from the methyl end. Data are the means of three measurement replicates from one independent experiment (*n* = 3). Error bars represent standard deviations.

As the largest fraction of the algal lipidome (Horn and Benning, 2016), glycerolipids are specifically distributed into distinct subcellular membranes that execute unique functions (Moellering and Benning, 2011) and possess characteristic fatty acyl compositions (Trémolières, 1998). Therefore, the acyls of glycerolipids were analyzed for both the chloroplast and the entire cell. Four special fatty acids, i.e., 18:3n6, 18:4n3, 16:4n3, and 18:3n3 (number of carbons:number of double bonds with n-number, which indicates the position of the double bond from the methyl end), were found to be distributed in the chloroplast with distinct proportions (**Figures 4A**). The chloroplasts isolated under higher rotation speeds (Groups N + 2, 4, and 6) contained lower proportions of 18:3n6 (40, 40, and 38%) and 18:4n3 (57, 66, and 56%) of the whole cells (**Table 2**). In contrast, these chloroplasts had relatively higher proportions of 16:4n3 (86, 91, and 92%) and 18:3n3 (83, 88, and 88%). In particular, 18:3n6 and 18:4n3 are mainly limited to DGTS (diacylglycerol-*N, N, N*-trimethylhomoserine) and PE (phosphatidylethanolamine), which are the presumed signature lipids of extra-plastidic membranes, especially the ER (Giroud et al., 1988; Trémolières, 1998); 16:4n3 and 18:3n3 are exclusively enriched in MGDG (monogalactosyldiacylglycerol) and DGDG (digalactosyldiacylglycerol), which are the postulated signature lipids of the chloroplast (Giroud et al., 1988; Moellering and Benning, 2011). Based on the proposed principles (Giroud et al., 1988; Trémolières, 1998; Moellering and Benning, 2011), 18:3n6 and 18:4n3 can serve as negative biomarkers and 16:4n3 and 18:3n3 positive biomarkers of the isolated chloroplasts. In this study, their distribution ratios in the chloroplast were considered to represent the purity and integrity of this organelle in *C. reinhardtii*. Thus, the chloroplasts of Groups N + 2, 4, and 6 (**Table 1**) were presumed to contain more MGDG and DGDG and less DGTS and PE, indicating that these chloroplasts were of relatively higher integrity and purity. To further identify chloroplasts with higher quality, the mass ratios of (16:4n3 + 18:3n3) to (18:3n6 + 18:4n3), indicated as *R*, were compared. The results showed that the *R*-values of chloroplasts obtained from distinct isolation conditions were all greater than 7 and at least 1.5-fold of that in the whole cell, showing notable differences between the chloroplast and the whole cell. The highest *R*-value was observed for chloroplasts isolated under the highest pressure and centrifugation speed (Group N + 6, **Table 1**), reaching 9.89, which was twofold of that in the whole cell and assumed to be the chloroplasts with the best quality. In addition, these chloroplasts exhibited the weakest band of BIP, an ER marker, by western blotting, showing the lowest amount of ER (**Figure 4B**). Under transmission electron microscopy, the chloroplast was found to be morphologically intact, enclosed in the envelope membrane and with the central pyrenoid encircled by a starch sheath. Thus, high-quality chloroplasts had less than 38% of 18:3n6 and more than 88% of both 16:4n3 and 18:3n3 of that in the whole cell.

**Table 2:** The percentage (%) of fatty acid biomarkers of chloroplasts in whole cells under normal conditions.

Fatty acid	Percentage (%)				
	N + 1	N + 2	N + 3	N + 4	N + 5	N + 6
18:3n6	50 ± 3	40 ± 2	59 ± 4	40 ± 2	45 ± 2	38 ± 2
18:4n3	64 ± 3	57 ± 3	78 ± 4	66 ± 4	80 ± 3	56 ± 3
16:4n3	85 ± 2	86 ± 5	99 ± 1	91 ± 5	89 ± 5	92 ± 5
18:3n3	83 ± 3	83 ± 2	96 ± 4	88 ± 4	86 ± 4	88 ± 4

*The percentage of fatty acid biomarkers of chloroplasts in whole cells is calculated as “(the mass of fatty acyl group of chloroplasts) / (the mass of fatty acyl group of the whole cells) × 100%” according to the data of **Figure 4A**. N + 1, N + 3, and N + 5 refer to normal chloroplasts isolated after exposure to 0.35, 0.55, and 0.75 MPa, respectively, under the same rotation speeds of 750 × *g* and subsequently 670 × *g*. N + 2, N + 4, and N + 6 refer to normal chloroplasts isolated after exposure to 0.35, 0.55, and 0.75 MPa, respectively, under the same rotation speeds of 5000 × *g* and subsequently 5000 × *g*. The fatty acid is expressed as number of carbons:number of double bonds with n-number, indicating the position of the double bond from the methyl end. Data are mean ± standard deviation of three measurement replicates from one independent experiment (*n* = 3).*

### Chloroplast Isolation from Cells under Nitrogen Stress

**FIGURE 5:**
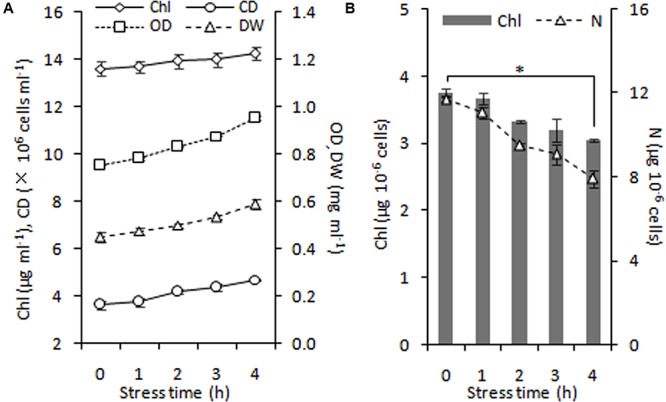
Time course of growth performance of *C. reinhardtii* under nitrogen stress. **(A)** Changes in cell growth parameters in response to nitrogen stress. **(B)** Levels of intracellular chlorophyll (Chl) and nitrogen (N) content under nitrogen starvation. An asterisk denotes significant (*P* < 0.05) decreases of the cellular Chl and nitrogen content within 4 h of nitrogen stress. Data are the means of three independent experiments (*n* = 3). Error bars represent standard deviations. CD, OD and DW denote cell density, optical density at 750 nm and cellular dry weight.

During 4 h of exposure to nitrogen stress, there was a slight increase in cell density from 3.61 to 4.68 × 10^6^ cells ml^-1^ (OD_750_ increased from 0.752 to 0.950), and the cellular dry weight density also showed an elevation from 0.45 to 0.59 mg ml^-1^ (**Figure 5A**). The cell dry weight remained unaltered (∼120 μg per million cells). The Chl concentration did not alter significantly, albeit exhibited a slight tendency to increase from 13.60 to 14.24 μg ml^-1^. These gradual variations indicated that cell division occurred in the early stage of nitrogen stress. The Chl and nitrogen content of each cell decreased by 19% (calculate using Chl concentration and cell density) and 32% (**Figure 5B**), respectively, representing the stress levels of the algal cells. Further, the six conditions designed for the normal chloroplast isolation were applied to prepare nitrogen-stressed chloroplasts. Only 0.5–3% of chloroplasts were successfully isolated from stress-treated cells, a sharp decline of 80∼98% compared with normal chloroplasts (**Figure 2A, Table 3**), which indicated that the N-starvation conditions could only be imposed for at most 4 h. Thus, 4 h was set as the maximum limited stress duration for successful chloroplast isolation. In addition, the chloroplast yield from stress-induced cells was highest at low speeds (750 × *g* for 2 min and 670 × *g* for 1 min) (**Table 3**), which was opposite to that under normal conditions. It is likely that when the stronger centrifugation force acted on the less resistant cells, the chloroplast membranes were more easily destroyed, severely hindering isolation of stressed chloroplasts.

**Table 3:** Distinct yields and fatty acid methyl ester mass ratio (R) of chloroplasts isolated from *C. reinhardtii* under nitrogen stress.

Group	Condition	Yield (%)	*R*
N - 1	0.35 MPa; 750 × *g*, 2 min; 670 × *g*, 1 min	2.66 ± 0.11	8.97 ± 0.47
N - 2	0.35 MPa; 5000 × *g*, 2 min; 5000 × *g*, 1 min	0.59 ± 0.04	nd
N - 3	0.55 MPa; 750 × *g*, 2 min; 670 × *g*, 1 min	3.06 ± 0.14	9.24 ± 0.43
N - 4	0.55 MPa; 5000 × *g*, 2 min; 5000 × *g*, 1 min	2.00 ± 0.15	10.49 ± 0.57
N - 5	0.75 MPa; 750 × *g*, 2 min; 670 × *g*, 1 min	2.09 ± 0.16	11.29 ± 0.54
N - 6	0.75 MPa; 5000 × *g*, 2 min; 5000 × *g*, 1 min	0.48 ± 0.03	nd
N - cell	nd	nd	4.23 ± 0.28

**FIGURE 6:**
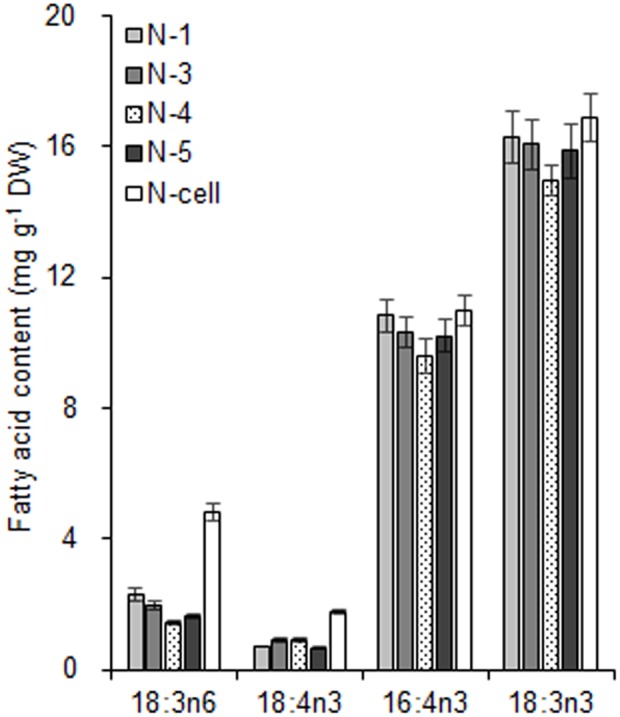
The fatty acid biomarkers of chloroplasts isolated from *C. reinhardtii* in response to nitrogen stress. N-1, N-3, and N-5 refer to stressed chloroplasts isolated after exposure to 0.35, 0.55, and 0.75 MPa, respectively, under the same rotation speed 750 × *g* and subsequently 670 × *g*. N-4 refers to stressed chloroplasts isolated after exposure to 0.55 MPa, under 5000 × g and subsequently 5000 × g. N-cell refers to cells cultured under nitrogen stress. The cellular fatty acid content is denoted as mg of fatty acyl groups per g of the cellular biomass (DW). The mass of chloroplast fatty acid was normalized to the original cellular dry biomass, which was based on the chlorophyll (Chl) amount, and denoted as mg of fatty acids per g of the equivalent cellular dry biomass. The fatty acyl is expressed as number of carbons:number of double bonds with n-number, indicating the position of the double bond from the methyl end. Data are the means of three measurement replicates from one independent experiment (*n* = 3). Error bars represent standard deviations.

*N - 1, N - 2, N - 3, N - 4, N - 5, and N - 6 refer to stressed chloroplasts isolated from six conditions. N-cell refers to cells cultured under nitrogen stress. The yield is calculated as “(chlorophyll content of isolated chloroplasts)/(chlorophyll content of the corresponding cells) × 100%”. *R* means the mass ratio of (16:4n3 + 18:3n3) to (18:3n6 + 18:4n3) and is calculated using the data of **Figure 6**. nd, not detected. Data are mean ± standard deviation of three measurement replicates from one independent experiment (*n* = 3).*

With respect to the proposed criteria involving the fatty acid biomarkers under normal conditions, the fatty acyl profiles of stressed chloroplasts were further analyzed for quality evaluation (**Figure 6**). Due to the limitation of the minimum amount required for total fatty acid analysis (Liu et al., 2015), the fatty acyl compositions of chloroplasts from Groups N-2 and 6 were not determined. Lipid analysis showed that the isolated Group N-5 chloroplasts contained the lowest levels of negative fatty acid biomarkers (no more than 38% for both 18:3n6 and 18:4n3) and relatively high levels of positive fatty acid biomarkers (more than 90% for both 16:4n3 and 18:3n3; **Table 4**), resulting in the highest *R*-value (**Table 3**), which was postulated to be the chloroplasts with the best quality. Further, the quality of these chloroplasts were validated using Western blot and morphology observation. The stressed chloroplasts isolated from Group N-5 contained very low levels of ER, as revealed by the BIP marker test (**Figure 2B**) and retained good integrity as the normal chloroplasts (**Figure 2C**). Nevertheless, these chloroplasts were disorganized to some extent and exhibited less compactness, with some reduction of appressed regions of the thylakoids, which was consistent with the decrease in the Chl content of the stressed cells. In addition, starch granules significantly accumulated and were scattered among the thylakoid membranes, leading to the chloroplast being distorted and obscured. Taken together, sufficient amounts of chloroplasts were successfully isolated from *C. reinhardtii* cells following 4 h of nitrogen stress, and their purity and integrity remained uncompromised.

**Table 4:** The percentage (%) of fatty acid biomarkers of chloroplasts in whole cells under nitrogen stress.

Fatty acid	Percentage (%)			
	N - 1	N - 3	N - 4	N - 5
18:3n6	48 ± 2	41 ± 1	30 ± 2	34 ± 2
18:4n3	41 ± 1	51 ± 2	52 ± 2	38 ± 2
16:4n3	99 ± 4	94 ± 3	87 ± 3	93 ± 4
18:3n3	96 ± 3	95 ± 4	89 ± 3	94 ± 3

*The percentage of fatty acid biomarkers of chloroplasts in whole cells is calculated as “(the mass of fatty acyl group of chloroplasts)/(the mass of fatty acyl group of the whole cells) × 100%” according to the data of **Figure 6**. N-1, N-3, and N-5 refer to stressed chloroplasts after exposure to 0.35, 0.55, and 0.75 MPa, respectively, under the same rotation speed 750 × *g* and subsequently 670 × *g*. N-4 refers to stressed chloroplasts isolated after exposure to 0.55 MPa, under 5000 × *g* and subsequently 5000 × *g*. The fatty acid is expressed as number of carbons:number of double bonds with n-number, indicating the position of the double bond from the methyl end. Data are mean ± standard deviation of three measurement replicates from one independent experiment (*n* = 3).*

## Discussion

In response to nitrogen stress, the *C. reinhardtii* chloroplast plays a crucial role in TAG accumulation (Du and Benning, 2016; Xu et al., 2016). However, the contribution by chloroplasts in comparison to the ER with regard to TAG accumulation remains veiled. To help resolve this issue, it is imperative to obtain chloroplasts with high quality.

### Homogenous Cultivation in PBRs Contributes to Reproducible Isolation of Chloroplasts

In previous studies, autotrophic or mixotrophic algal cultures are normally maintained in flasks, and the required volumes for chloroplast isolation are reportedly no more than 1 – 2 L (Bennoun, 1982; Bollivar and Beale, 1995; Mason et al., 2006). However, the culture volume for stressed chloroplast isolation is much higher than that under normal conditions because of the much lower isolation yield. In this case, cultivation in PBRs is a feasible approach to provide an adequate volume of stressed cells for chloroplast isolation. In the current study, uniform algal populations were obtained from cyclic subcultures in flasks and stable batch cultures in PBRs, which is vital to yielding abundant chloroplasts in similar physiological state. To a large extent, this process depends on a constant transferring cycle of liquid subculture in flasks (7 days in this study) under a synchronous light-dark cycle. In addition, *C. reinhardtii* propagates more slowly in minimal medium, even when supplemented with CO_2_, compared with TAP medium, which contributes to the formation of a uniform culture. This autotrophic culture mode is able to prevent bacteria from frequent reproduction (Mason et al., 2006), especially at the initial growth phase when the cell density is low, i.e., ∼10^4^ cells ml^-1^ (**Figure 3**). Axenic culture derived from agar-solidified medium is also crucial to ensure vigorous growth of cells in flasks and PBRs.

The culture system in PBRs is stable and favorable for obtaining reproducible batch cultures. Moreover, the cell doubling time was notably reduced when the cultures were transferred into PBRs aerated with 4% CO_2_ (see Materials and Methods). In this case, more chloroplasts could be isolated from more algal cultures in PBRs at one time, which is a time- and space-saving approach compared to flask culture. Based on a 500 ml algal culture containing 10 mg Chl, the stressed chloroplast yield was 0.5∼3%, containing 50 – 300 μg Chl and no more than 530 μg FAMEs (Supplemental Table S1). Chloroplast isolation under stress conditions largely relies on cyclic and stable pre-cultivation in flasks and PBRs, which serves as an essential assurance for isolating more homogenous chloroplasts.

### Cell Disruptor with Gentle Mechanical Shear Force is Vital to Keeping Chloroplast Intact

In addition to chemical lysis (Klein et al., 1983; Kreuzberg et al., 1987; Sultemeyer et al., 1988), mechanical methods through nitrogen decompression are often utilized to break cells for chloroplast isolation from *C. reinhardtii*. Although the Yeda press was widely used in the 1980s (Belknap and Togasaki, 1981; Mendiola-Morgenthaler et al., 1985; Goldschmidt-Clermont et al., 1989), it had less satisfactory results for cells grown photoautotrophically and tended to result in more ruptured chloroplasts (Moroney et al., 1987; Mason et al., 1991). Subsequently, a Bioneb nebulizer, available since the 1990s, became an alternative device for breaking cells (Bollivar and Beale, 1995; Jans et al., 2008; Burgess et al., 2016). In addition, a few custom-made devices (Naumann et al., 2005; Mason et al., 2006; Terashima et al., 2010) have been used to break *C. reinhardtii* cells. When Bioneb was applied to nitrogen-starved cells in our preliminary experiments, its mechanical shear force appeared to be so strong that we observed reduced integrity of stressed chloroplasts. Thus, a simple mechanical disruptor was designed (**Figure 1B**) to gently break stress-treated cells and release intact chloroplasts without strong mechanical force. Moreover, this device can be easily assembled and modified as needed. Its principle depends on nitrogen decompression. First, a massive amount of nitrogen is dissolved in concentrated cells in a stainless steel vessel under a designated pressure. When the gas pressure is controllably released from the needle valve, the nitrogen separates from the cells as expanding bubbles that stretch the membrane of each cell until they rupture, and multiple cell fragments including chloroplasts are then released from the disruptor. As this disruption device requires no electricity and generates no heat, the concentrated cells can be chilled to protect them from damage. The available volume of the cylinder tube body is 10 – 40 ml, and at most 15 mg Chl-containing cells can be broken at one time.

In this study, this custom-made disruptor produced 5 – 30% of normal chloroplasts (ranges of twelve independent tests), consistent with previous reports (Klein et al., 1983; Katzman et al., 1994; Mason et al., 2006). More importantly, it also generated 0.5 – 3% of stressed chloroplasts (ranges of seven independent tests). As an environmental sensor (Chan et al., 2016), the nitrogen-stressed chloroplasts became distorted, as determined by an decrease in appressed regions of the thylakoids and an increase in starch granules (**Figure 2C**), which were prone to result in broken chloroplasts and thought to be key limiting factors for yielding large amounts of intact chloroplasts (Klein et al., 1983; Fan et al., 2011). The physical features of the cell slurry, such as viscosity, cell density, particle size and settleability of the suspension, jointly affect the disruption level of the cells (Fleischer and Rouser, 1965; Chisti and Mooyoung, 1986). Additionally, the cell disruption process should be rapid; otherwise, the extruded chloroplast from a cell is likely to be ruptured (Moroney et al., 1987). It is suggested that a cell disruptor with similar principles of mild mechanical shear force on cells would be appropriate to gently break stress-induced cells and maintain good integrity of the chloroplast.

### A Quality Control Method Involving the Fatty acid Biomarkers Is Feasible

Due to the different target applications of the isolated chloroplasts, the quality criteria also differ. Normally, the quality of *C. reinhardtii* chloroplasts involves three aspects, i.e., yield, purity and integrity (Mason et al., 2006). The yield is based on Chl recovery, and the purity or contamination depends on the presence of marker proteins in other sub-compartments, especially the mitochondria and cytosol, which are commonly less than 20% (Belknap, 1983; Klein et al., 1983; Kreuzberg et al., 1987). The fraction of isolated chloroplasts in this study was expected to be free of ER, the other essential organelle for TAG accumulation. Immunoblotting analyses revealed the slight ER contamination revealed by BIP detection, which were also reported in a previous study (Warakanont et al., 2015). Integrity is assessed by oxygen evolution using a ferricyanide assay, which is often more than 80% (Belknap, 1983; Kreuzberg et al., 1987). Furthermore, electron microscopy is often used to confirm the purity and integrity of chloroplasts (Klein et al., 1983; Mendiola-Morgenthaler et al., 1985; Mason et al., 2006). In our study, electron microscopy images showed that both the normal and stressed chloroplasts were largely intact. However, because of our aim to study subcellular lipid metabolism in *C. reinhardtii*, it was necessary to focus on chloroplast quality in terms of the lipid composition.

According to the proposed criteria based on the fatty acid biomarkers, the isolated chloroplasts with fewer proportions of 18:3n6 and 18:4n3 mainly in DGTS and PE and greater proportions of 16:4n3 and 18:3n3 mainly in MGDG and DGDG were likely to be less contaminated by ER and having relatively intact envelopes. The proposed criteria were deduced from a general principle, whereby the preferential distribution of fatty acids at the sn-2 position of glycerolipids reflects their biosynthetic origin from the ER or chloroplast in plants (Roughan and Slack, 1982). Based on that, the chloroplast of *C. reinhardtii* was presumed to contain 14% DGTS, 11% PE, 97% MGDG and 97% DGDG (Giroud et al., 1988), indicating that a majority of DGTS and PE reside in the ER and that most MGDG and DGDG are present in the chloroplast of *C. reinhardtii*. However, what is the lowest proportion of 18:3n6 and 18:4n3 of DGTS and PE in ideal pure chloroplasts of *C. reinhardtii*? Janero and Barrnett (1981) detected 38% DGTS in chloroplast membranes. In this study, the 18:3n6 and 18:4n3 levels of DGTS and PE were no more than 38% and 16:4n3 and 18:3n3 levels of MGDG and DGDG were greater than 88% in the best quality chloroplasts (Groups N + 6 and N - 5) under normal and stress conditions, which were all close to the reported results. In addition, the immunoblotting analysis and ultrastructure observation further verified the purity and integrity of these chloroplasts. Therefore, it is feasible to monitor chloroplast quality using the fatty acid biomarkers, which are specifically localized in distinct subcellular compartments.

### Stringent Culture Conditions and Prompt, Rapid Operations Facilitate Successful Chloroplast Isolation

Successful isolation of high-quality chloroplasts largely depends on stringent culture conditions and prompt, rapid operations. First, there are two vital points regarding the culture conditions. It is important to maintain a uniform culture through cyclic and stable subcultures in flasks and PBRs along with a synchronous light cycle, which can further facilitate uniform application during stress cultivation. Note that the degree of stress is equally critical for successful isolation, including the induction time, light intensity, inoculation density and other stress factors. It is critical to initially optimize the degree of stress, as excessive stress treatment can cause the cells to rupture more completely, thus making it more difficult to obtain intact chloroplasts. Second, the entire procedure of chloroplast isolation should be conducted quickly, consecutively and promptly. Once the concentrated cells are disrupted, any unnecessary delays can lead to distinct aggregation, which can lead to clump formation and disruption of the Percoll gradient, hindering the correct loading of subfractions into the corresponding gradient. It is also vital to resuspending the pellet gently and thoroughly using a fine paint brush. Insufficient resuspension is likely to result in aggregates, which can also prevent successful isolation.

## Conclusion

In this study, starting with a 500 ml culture containing 10 mg Chl, stressed chloroplasts with 48 – 300 μg Chl were successfully isolated from *C. reinhardtii* cells. These chloroplasts appeared to be intact, as determined by the fatty acid biomarker analysis and ultrastructure observation. Homogenous batch cultures in PBRs and gentle cell disruption enabled the reproducible isolation of stressed chloroplasts. To the best of our knowledge, this is the first report of successful chloroplast isolation from microalgal cells under stress conditions, which can provide insight into the nature of stressed chloroplasts and offer available biomaterials for study of subcellular lipid metabolism in microalgae. Future work is necessary to isolate chloroplasts from microalgal cells following longer period of abiotic stress, which can facilitate understanding of subcellular biochemistry and biology of microalgae.

## Author Contributions

MY and SX conceived and designed the research. MY and J-PJ performed the experiments and analyzed data. MY, XX, and SX wrote the manuscript. Y-DC designed the cell disruptor. Y-DC and X-PC provided technical assistance. YF participated in algal cultivation. XX and Z-YC supervised specific experiments. All authors agreed on the manuscript.

## Conflict of Interest Statement

The authors declare that the research was conducted in the absence of any commercial or financial relationships that could be construed as a potential conflict of interest.

## Footnotes

1 http://www.chlamycollection.org/

2 http://www.scbt.com

3 http://www.beyotime.com
