# Supplementary material for: Chloroplasts Isolation from Chlamydomonas reinhardtii under Nitrogen Stress
Source: Front Plant Sci. 2017 Aug 29;8:1503. doi: 10.3389/fpls.2017.01503 (PMC5581827; doi:10.3389/fpls.2017.01503)
Supplement: Supplementary file 1 [file Table_1.DOCX]

**Supplemental Table S1** **The mass of fatty acid methyl esters (FAMEs) prepared from isolated chloroplasts and cells under nitrogen stress.** N-1, N-3 and N-5 referred to the stressed chloroplasts from 0.35, 0.55 and 0.75 MPa, respectively, under the same rotation speed 750 g and subsequent 670 g. N-4 referred to stressed chloroplasts isolated from 0.55 MPa, under 5000 g and subsequent 5000 g. N-cell referred to cells cultured under nitrogen stress. The mass of each chloroplast and cellular fatty acyl groups were normalized to chlorophyll mass. Values represented the mean ± standard deviation of three measurement replicates from one independent experiment (n=3). FAMEs and Chl are fatty acid methyl esters and chlorophyll.

| Group | N-1 | N-3 | N-4 | N-5 | N-cell |
| --- | --- | --- | --- | --- | --- |
| FAMEs (μg mg^-1^ Chl) | 1844±89 | 1741±78 | 1621±67 | 1644±79 | 2163±82 |
